# Supplementary material for: Recall, understanding, use, and impact of front-of-package warning labels on ultra-processed foods: A qualitative study with mothers of preschool children in Peru
Source: PLOS Glob Public Health. 2024 Dec 12;4(12):e0003938. doi: 10.1371/journal.pgph.0003938 (PMC11637309; doi:10.1371/journal.pgph.0003938)
Supplement: Attachment — Submitted filename: Response to Reviewers 05.09.2024.pdf [file pgph.0003938.s001.pdf]

Response to Reviewers

Lima, September 5th 2024

Manuscript Number: PGPH-D-24-01396
Title: Recall, understanding, use, and impact of front-of-package warning labels on ultra-processed foods: A qualitative study with mothers of preschool children in Peru.

Dear editor,

We would like to thank for reviewing our manuscript entitled "Recall, understanding, use, and impact of front-of-package warning labels on ultra-processed foods: A qualitative study with mothers of preschool children in Peru" and for the opportunity to provide response to the feedback received.

We wish to express our gratitude to the editor and the reviewer for their time invested in reading our manuscript. We are thankful for their suggestions and comments, which will result in an improved version of the manuscript.

Please find below our point-by-point response to all the comments provided. All changes have been included in the manuscript using track changes.

We look forward to hearing from you.

Sincerely,

Francisco Diez-Canseco on behalf of the authors

Reviewer #1: The article is interesting because it could contribute to Peru's current policy on front-of-package labeling. However, I must highlight some points in the article that need to be adjusted in a major way. I list these points according to the position of the line:

Response. *We are grateful for the reviewer's comments and suggestions, which we have answered one by one, and used to improve the manuscript.*

line 76: The phases of implementation of labeling are described to allow the industry to adapt, and the table shows the profiles of each stage. However, this article does not discuss changes between implementation phases or evaluate seals between products. I suggest considering avoiding and/or removing paragraph and table 1.

Response. *Thanks for the suggestion. Although the study does not address the phases of implementation as an object of research, the brief text included in the introduction provides a basic idea of the implementation of the policy in Peru -specially relevant for non-Peruvians- and gives context to the information gathered. We agree that the table could be too much detail, and we have removed it from the manuscript.*

line 113: It is mentioned that participants were selected from a national household survey. However, the reference of the survey is not detailed or placed, what year is it, who made it, etc.? It also does not detail how the women were selected, was it random, was it for convenience, for example, what was the total sample and which was the selected one, how were they contacted? what was done in case they were not contacted? What was the positive response rate? etc.

Response. *The participants were not selected from the sample of the National Household Survey. Our study used the SES classification of the Peruvian National Household Survey (that has 5 different strata, being "A" the highest to "E" the lowest) to select women from two different SES ("B" or middle-high" and "D" or middle-low). We have clarified this in the manuscript to avoid confusion.*

Additionally, we have expanded the description of the recruitment processes of the study participants in the "Procedures" section of the manuscript, including the number of women invited and those who finally accepted.

line 117: It states the variables that were used to evaluate SES, could you please state what was the basis or reference of how the SES was constructed for the selection?

Response. *Thank you for your query. At the end of the sentence describing the variables used to estimate the participants' SES in our study, we have included the reference to a document of the Peruvian Association of Marketing Companies, where the methodology is described. In our study, all the potential participants answered a short questionnaire that collects all these variables and classifies them, based on their responses, into one of the 5 posibles SES. Those who were assigned to SES B or SES D, and met the other study inclusion criteria, were invited to participate.*

line 130: Data collection tools. Table 2 describes the themes used in the focus group guide.

However, the methodology does not explain whether any model, framework or theory was used to measure the themes of recall, use, change of purchase, perception or judgment. That is, how these variables were defined in order to develop the guide. Also, it does not detail how the guide was developed. Was it constructed? or adapted from one already developed and validated?

Response. *Thanks for your question. The development of the focus group guide was not based on a framework or adapted from a previous guide, but developed specifically for this study, based on the topics prioritized by the research team. The pertinence of the guide's topics and questions, as mentioned in the manuscript, were then tested and verified in two pilot focus groups. We have now included in the manuscript that the guide was developed specifically for the study.*

line 134: The use of images is mentioned, 1) could you explain why these were used, 2) which category of UPP was used and why, 3) what type of beverage, perhaps it would be worthwhile to provide examples of these.

Response. *Thank you for your request. In the manuscript, we have expanded the explanation of why the images were used to support the conversation. In addition, we have added a brief description of the image of the processed products, which in fact included 3 photographs with various different packaged beverages and foods, to prevent participants from focusing on only a few of them during the conversation (we have included the image in this letter to inform our reviewer).*

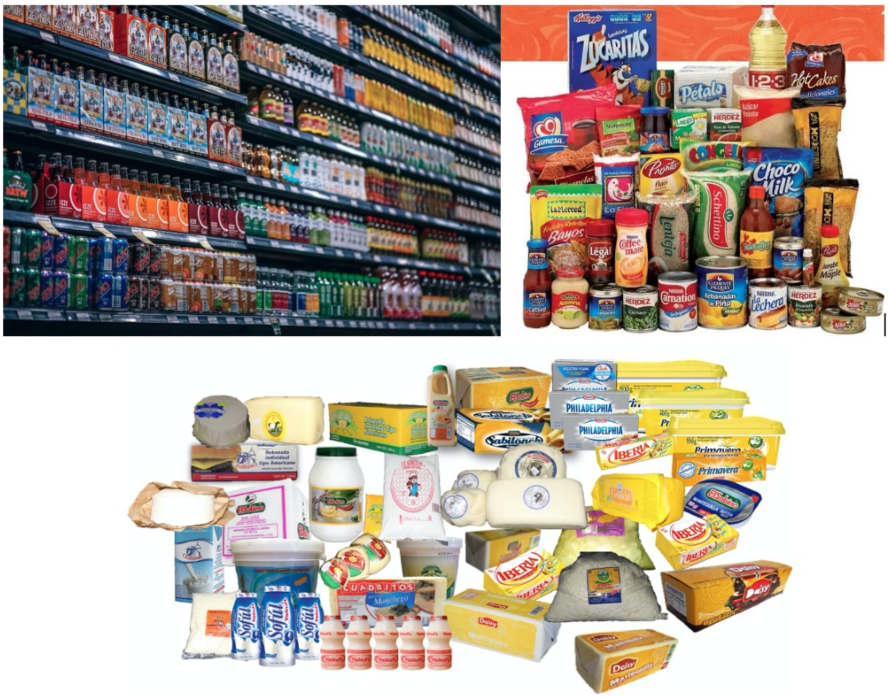

line 147: describe the profile and experience of the person who conducted the focus groups.

Response. *Thanks for the suggestion. We have included a line to describe the background and experience of the researcher conducting the focus groups.*

line 157: describe what type of analysis was performed: content, thematic, narrative, grounded theory or discourse analysis?

Response. *The manuscript already mentions that a content analysis was conducted (line 165 of the manuscript with track changes).*

line 177: Table 3 does not describe the mothers' level of education (an important variable for the study) or the sociodemographics of the children as described in lines 136-138.

Response. *Thanks for the request. We have included the mothers' education level and the gender and age of their children in Table 2 (Table 3 in the original manuscript).*

Line 172: The subtitles of the results do not coincide with the topics described in table 2, for example, topic 5 (table 2) is not mentioned and others seem to have been edited for convenience.

Response. *Thanks. We have included most of the subheadings of the "Results" section in Table 2 for consistency.*

Line 386: Much of the discussion is devoted to discussing a summary of the results. It is compared with a systematic review and a study from Chile, however due to the magnitude of Latin America countries that have implemented FOP warnings there is a need for a comparison at the same level. There are studies from Brazil, Canada and Mexico with a qualitative character that could be discussed.

Response. *Thanks for the suggestion. We have reviewed studies from Mexico and Brazil focused on the understanding, perceptions and use of the FOPWL and discussed some of their findings in the 4th and 6th paragraph from the "Discussion" section.*

line 475: I think more can be said about strengths and limitations. For example, the sample was obtained secondarily from a national survey and was not specifically identified for this study, such as education, and regular consumers, but specific inclusion criteria were identified that could have a homogeneous geographic and SES distribution.
Or for example, that although possible they did not remember after two years but were used example images of the products. Or for example, self-reported use or purchase may be influenced by educational level or the presence of previous chronic diseases (the latter was not included), etc.

Response. *Thanks for the suggestions. In our response to the reviewer's 2nd comment, we clarified that the sample was specifically recruited for this study -not from a national survey. We have explained that in the reviewed manuscript. Regarding the possibility of recall bias,*

we agree, and this is mentioned in the “Strengths and limitations” section. We have added as a new limitation that the remembering and use of the warning labels and the purchase changes could be linked to variables we did not consider and collect during the recruitment, such as the presence of a chronic condition in the household.

Thanks again for the comments and suggestions.
